# Changes in Consumer Attitudes toward Broad-Based and Environment-Specific Sodium Policies—SummerStyles 2012 and 2015

**DOI:** 10.3390/nu9080836

**Published:** 2017-08-04

**Authors:** Erika C. Odom, Corine Whittick, Xin Tong, Katherine A. John, Mary E. Cogswell

**Affiliations:** 1 Epidemiology and Surveillance Branch, Division for Heart Disease and Stroke Prevention, National Center for Chronic Disease Prevention and Health Promotion, Centers for Disease Control and Prevention, 4770 Buford Hwy NE, Atlanta, GA 30341, USA; fvx4@cdc.gov (X.T.); yfr6@cdc.gov (K.A.J.); mec0@cdc.gov (M.E.C.); 2 United States Public Health Service, Commissioned Corps, Rockville, MD 20852, USA; 3 Mailman School of Public Health, Columbia University, New York, NY 10032, USA; corinewhittick@gmail.com; 4 Project IMHOTEP, Morehouse College, Atlanta, GA 30314, USA; 5 IHRC, Inc., Atlanta, GA 30346, USA

**Keywords:** attitudes, sodium reduction, policies, consumer

## Abstract

We examined temporal changes in consumer attitudes toward broad-based actions and environment-specific policies to limit sodium in restaurants, manufactured foods, and school and workplace cafeterias from the 2012 and 2015 SummerStyle surveys. We used two online, national research panel surveys to conduct a cross-sectional analysis of 7845 U.S. adults. Measures included self-reported agreement with broad-based actions and environment-specific policies to limit sodium in restaurants, manufactured foods, school cafeterias, workplace cafeterias, and quick-serve restaurants. Wald Chi-square tests were used to examine the difference between the two survey years and multivariate logistic regression was used to obtain odds ratios. Agreement with broad-based actions to limit sodium in restaurants (45.9% agreed in 2015) and manufactured foods (56.5% agreed in 2015) did not change between 2012 and 2015. From 2012 to 2015, there was a significant increase in respondents that supported environment-specific policies to lower sodium in school cafeterias (80.0% to 84.9%; *p* < 0.0001), workplace cafeterias (71.2% to 76.6%; *p* < 0.0001), and quick-serve restaurants (70.8% to 76.7%; *p* < 0.0001). Results suggest substantial agreement and support for actions to limit sodium in commercially-processed and prepared foods since 2012, with most consumers ready for actions to lower sodium in foods served in schools, workplaces, and quick-serve restaurants.

## 1. Introduction

Excessive consumption of dietary sodium is a major risk factor for hypertension, and subsequent heart disease and stroke, two of the leading causes of death in the United States. The average daily intake of sodium among U.S. adults is about 3500 mg/day [1], excluding salt added at the table, which far exceeds recommendations to limit sodium intake (<2300 mg/day) [2]. Although nearly half of adults report taking actions to reduce their sodium intake [3], voluntary initiatives that focused on individual sodium education and behaviors have not significantly lowered population sodium intake [4]. In fact, about 90% of U.S. adults still consume too much sodium [4]. Because most of the sodium consumed comes from commercially-processed and prepared foods [5], in 2010, the Institute of Medicine recommended government action to reduce sodium in the U.S. food supply. Reducing sodium calls for a multifaceted approach that includes the collaboration of food manufacturers, industry/vendors, and local policies [6,7,8]. Consumer agreement with such broad-based actions could suggest support for sodium reduction in manufactured and prepared foods.

In 2010, most respondents to a nationwide survey supported broad-based actions or environment-specific policies to limit sodium in manufactured foods (55.9%), restaurant food (47.0%), and food served in quick-serve restaurants (81.5%) [9]. Recent surveys from 2010 and 2012 suggests that at least 80% of U.S. adult consumers would support national standards that limit sodium in foods served in school cafeterias [10,11]. In a 2013 study, about half of respondents indicated support for policies increasing healthy food and drink options served in workplace cafeterias and vending machines [12]. Data also suggests that the promotion of low-sodium food options in the workplace may increase consumer’s acceptance and willingness to choose healthier, low-sodium options while at work [13,14]. To better understand changes in consumer readiness for sodium-related policies, in this study we use data from the SummerStyles 2012 and 2015 surveys to (1) assess the percentage of adults who support broad-based actions and environment-specific policies to reduce sodium in restaurants, school and workplace cafeterias, and in manufactured foods, and (2) to determine if support has changed from 2012 and 2015, overall, and among population subgroups.

## 2. Materials and Methods

A cross-sectional study was conducted using data from two of Porter Novelli’s online HealthStyles surveys. Data was collected by GfK’s KnowledgePanel^®^ (GfK North American Headquarters, New York, NY USA), an online national panel of noninstitutionalized U.S. participants. Panelists are randomly recruited by probability-based sampling (using random digit dial and addressed-based sampling methods) to reach respondents regardless of whether they have landline phones or Internet access, and are continuously replenished to maintain approximately 55,000 panelists. If needed, households are provided with a laptop computer and access to the Internet. The initial wave of SpringStyles was sent to a random sample of panelists ages 18 or older, while the second wave (SummerStyles) was sent to a random sample of panelists who completed the initial wave. Respondents could earn up to 20,000 cash-equivalent reward points (approximately $20) and were eligible to win an in-kind prize through a monthly sweepstakes if they participated in both waves. The Centers for Disease Control and Prevention (CDC) suggested potential questions to include, while Porter Novelli determined the final questionnaire content. CDC licensed the results (responses to the questions) from Porter Novelli. Licensed data provided did not include personally identifiable information and was determined exempt by CDC Institutional Review Board (IRB).

In total, 4170 panelists aged ≥18 years completed the 2012 SummerStyles survey, with a response rate of 65% (of respondents from the 2012 SpringStyles). In the 2015 SummerStyles survey, there were 4127 panelists aged ≥18 years, with a response rate of 67% (of respondents from the 2015 SpringStyles). In both surveys, the samples were weighted for age, gender, race, household income, education, census region, metropolitan status, and prior Internet access.

In this study, we excluded 268 participants (2012: *N* = 122 and 2015: *N* =146) who had missing information on demographic and health characteristics and 184 participants (2012: *N* = 122 and 2015: *N* = 62) who had missing responses on sodium-related questions. The final sample included 7845 respondents; 3926 in 2012 and 3919 in 2015, respectively.

### 2.1. Measures

#### 2.1.1. Consumer Agreement with or Support for Broad-Based Actions to Limit Sodium in Foods

To assess the level of consumer agreement with broad-based actions to limit sodium in restaurants and manufactured foods, participants were asked about agreement with the following statements: (1) “I think it’s a good idea for the government to keep restaurants from putting too much salt in food” and (2) “I think it’s a good idea for the government to keep food manufacturers from putting too much salt in food”. A five-point Likert scale was used to record responses: 1 = strongly disagree; 2 = somewhat disagree; 3 = neither agree nor disagree; 4 = moderately agree; and 5 = strongly agree. Responses of strongly disagree, somewhat disagree, and neither agree nor disagree were grouped together and termed neutral/disagree. Responses of strongly agree and somewhat agree were grouped together and termed agree. To assess the level of consumer support for environment-specific policies to limit sodium in school cafeterias, workplace cafeterias, and quick-serve restaurants, participants were asked about level of support for the following: (1) “policies that lower sodium/salt content of foods in school cafeterias”, (2) “policies to limit the amount of sodium/salt of foods in workplace cafeterias”, and (3) “policies to limit the amount of sodium/salt in quick-serve restaurants”. A four-point Likert scale was used to record responses: 1 = strongly oppose; 2 = slightly oppose; 3 = slightly support; 4 = strongly support. Responses of strongly oppose and slightly oppose were grouped together and termed neutral/not support, and responses of slightly support and strongly support were collapsed into support.

#### 2.1.2. Demographic and Health Characteristics

Categorical variables were constructed for age (18–30 years, 31–50 years, and 51 years and older); sex; race-ethnicity (non-Hispanic White, non-Hispanic Black, Hispanic, and other); household income (<$15,000, $15,000–24,999, $25,000–39,999, $40,000–59,999 and ≥$60,000); education level (high school or less, some college, and college graduate or higher); and region (Northeast, Midwest, South, and West). Body mass index (BMI) was calculated from self-reported height and weight and categorized as normal (<25 kg/m^2^), overweight (25–30 kg/m^2^), and obese (≥30 kg/m^2^). Self-reported hypertension status was determined by participant response to the question, “During the past year, have you had (or do you currently have) any of these health conditions: high blood pressure?”

#### 2.1.3. Consumer Desire to Eat Less Sodium

Participants’ level of agreement with the statement “I want to eat a diet that is low in sodium/salt” was assessed with a five-point Likert scale. Response categories were: 1 = strongly disagree; 2 = somewhat disagree; 3 = neither agree nor disagree; 4 = somewhat agree; 5 = strongly agree. Responses of strongly disagree and somewhat disagree were grouped together and termed, no. Responses of strongly agree and somewhat agree were grouped together and termed, yes, and neither agree nor disagree was termed neutral.

### 2.2. Statistical Analysis

Data were weighted to match the U.S. Current Population Survey (CPS) proportions, 2011 and 2014, using nine factors: sex, age, household income, race or ethnicity, household size, education, census region, metro status, and prior Internet access. Wald Chi-square tests were used to determine the differences between 2012 and 2015 in respondent attitudes toward broad-based actions or policies related to sodium reduction among various sociodemographic characteristics. After pooling the two years of data together, we performed a multivariate logistic regression analysis to examine the association between respondent characteristics and agreement or support for policies to limit sodium across all environments after controlling for survey year (2012, 2015), age, sex, race/ethnicity, household income, education level, BMI, hypertension status, and desire to eat a low-sodium diet. Adjusted odds ratios (aORs) and 95% confidence intervals (CI) were obtained, and a two-tailed *p*-value less than 0.05 was considered statistically significant. All statistical analyses were performed using SAS version 9.3 (SAS Institute Inc., Cary, NC, USA).

## 3. Results

Among the total 7845 respondents, 77.3% were 31 years or older, 51.2% were female, 67.0% were non-Hispanic white, 51.1% earned at least $60,000, 58.1% had more than a high school education, and 36.8% lived in the South (Table 1). There were no statistical differences on age, sex, race, household income, education level, region, BMI, or hypertension status between SummerStyles 2012 and 2015. The percentage of respondents who responded “No” (2012: 15.8%, 2015: 12.0%) to wanting to eat a diet low in sodium decreased from 2012 to 2015 (*p* = 0.0004).

Between 2012 and 2015, consumer agreement with broad-based actions to limit sodium in restaurant and manufactured foods did not change significantly. A little less than half of respondents (2012: 45.7%, 2015: 45.9%, Table 2) agreed with limiting sodium in restaurant foods, and more than half of respondents (56.5% for both 2012 and 2015) agreed with limiting sodium in manufactured foods. The lack of change between 2012 and 2015 was consistent across respondent characteristics, with a few exceptions. There was a decrease in consumer agreement to limit sodium in manufactured food among respondents that earned <$15,000 (*p* = 0.04) and who were neutral about eating a diet low in sodium (*p* < 0.05).

Between 2012 and 2015, support for environment–specific policies to reduce sodium in food prepared in school cafeterias (2012: 80.0%, 2015: 84.9%, Table 3), workplace cafeterias (2012: 71.2%, 2015: 76.6%), and quick-serve restaurants (2012: 70.8%, 2015: 76.7%) significantly increased. Overall, most respondents supported policies to reduce or limit sodium in these food outlets and the support increased between 2012 and 2015, but this increase was not statistically significant in all population subgroups. Increased support was seen among the following subgroups: those ≥31 years old, both males and females, non-Hispanic whites, those with a household income of ≥$40,000, those with some college education or higher, those who live in the Northeast, South, or Midwest, those who have a BMI <25 or ≥30 kg/m^2^, non-hypertensives, or those who have a desire to eat a diet low in sodium. Among Hispanic respondents, there was an increased support specifically for policies limiting sodium in school cafeterias.

Multivariate logistic regression analyses showed that, in 2015, respondents were significantly more likely to support environment-specific policies limiting sodium in school cafeterias (aOR = 1.37, CI = 1.18–1.61, Table 4), workplace cafeterias (aOR = 1.30, CI = 1.14–1.49), and quick-serve restaurants (aOR = 1.34, CI = 1.17–1.53) than in 2012.

## 4. Discussion

Overall, while approximately half of consumers agree that it is a good idea to have broad-based actions limiting sodium in restaurants and in manufactured foods, attitudes did not differ between 2012 and 2015. Most consumers also support environment-specific policies that limit sodium in school cafeterias, workplace cafeterias, and quick-serve restaurants with a small, but statistically significant increase in agreement between 2012 and 2015. Consumer support for environment-specific policies limiting sodium in cafeterias and quick-serve restaurants were seen across a range of sociodemographic subgroups in both years, with the highest support observed among non-Hispanic blacks. Similarly, among Hispanic adults there was a high support for policies limiting sodium in school cafeterias, workplace cafeterias, and quick-serve restaurants, with a trend toward increased support between 2012 and 2015—though it did not reach statistical significance. A previous study suggests that non-Hispanic blacks and Hispanics are more likely to report taking action to reduce their sodium intake and are also more likely to report being told by a healthcare professional to do so [15], which may suggest an openness towards policies limiting sodium in the food supply.

Although the current findings show that there has been substantial agreement among consumers to limit sodium in commercially processed and prepared foods in various settings since 2012, the percent agreeing with “government actions” to limit sodium was up to 40 points lower than the percent agreeing to support environment-specific “policies” to limit sodium. It is possible that survey respondents might be more likely to agree with or support questions when framed as a general policy rather than when framed as actions of the government. Yet, almost all regions around the world have government or industry-led strategies aimed at sodium reduction through the reformulation of manufactured foods [16]. Consumers may be more open to policy changes in specific settings or environments. Among all of the settings evaluated in this study, agreement to limit sodium was lowest for restaurants; however, agreement to limit sodium in quick-serve restaurants was up to 30 percentage points higher than agreement to limit sodium in all restaurants. Similar findings from 2010 HealthStyles data suggest that respondents might be more supportive of policies regulating the sodium content of quick-serve foods or fast food rather than those regulating all restaurants [9]. Given that U.S. adults consume up to one-third of their daily energy from away-from-home sources [17], and that processed foods (i.e., restaurant and manufactured foods) compose a majority of consumer sodium intake in the U.S. [5], more studies are needed to determine how consumer education/communication on the sodium content of foods in these environments may change sentiments in favor of sodium reduction policies.

Previous studies also suggest consumer readiness for sodium reduction in cafeteria foods. In a 2010 survey of U.S. consumers, 90% of respondents supported policies to lower sodium content in school cafeterias [10]. Likewise, a majority of respondents suggested willingness to support healthy food options, including reduced sodium foods in worksite cafeterias [12,14,18]. Public support of nutrition policies can bolster the implementation of local educational programs and environmental interventions, including sodium reduction efforts in schools and worksites.

To our knowledge, no prior study has examined changes over time in U.S. consumers’ attitudes about broad-based actions or policies to limit sodium across the range of food environments we examined. Most studies are limited to examinations at one point in time or include fewer food environments for addressing sodium reduction policies [9,13,19,20,21,22]. Our findings suggest increased support for environment-specific policies to lower sodium across most sociodemographic subgroups between 2012 and 2015, particularly those who were middle or older aged, non-Hispanic whites, earning ≥$40,000, college educated, not within normal BMI range, non-hypertensive, or who had a desire to eat a diet low in sodium. Although, some consumer groups appear ready to support policies to limit sodium in the food industry, the data from this study could be used to identify groups that could be targeted for interventional messaging and education on sodium reduction strategies from the medical community and public health agencies.

The findings presented in this study are subject to some limitations. The survey was not nationally representative of the U.S. population, although respondents were weighted to the general distribution of the U.S. population on age, sex, race/ethnicity, household income, household size, education, census region, metro status, and prior Internet access. Most of the respondents were middle or older aged, non-Hispanic whites, presenting an overrepresentation (in comparison to the general U.S. population), and had a household income ≥$60,000, which may impact the generalizability of the findings. Second, the limited sample size may have decreased statistical power to find a difference in some respondent subgroups. For example, the difference observed among Hispanics (14% of the total sample) in the percent who support sodium reduction in workplace cafeterias is larger in percentage points (i.e., 6.8 percentage points) than the statistically significant difference among non-Hispanic whites (6.4 percentage points, 67% of the total sample). However, the relative difference is smaller. Third, self-reported height and weight, which were used to calculate respondents’ BMI as well as hypertension status are subject to self-reporting bias. Respondents may have also provided socially desirable responses to questions on limiting sodium across various environments. Fourth, the results of the survey questions on limiting sodium which focus on “governmental actions” are not comparable to the questions that focus on “policies” due to the following: (1) participants’ perception of these terms may have elicited differences in their responses; (2) the settings rated by consumers differed between the governmental actions questions and the policies questions; (3) the response categories for questions on governmental actions included a neutral category (i.e., neither agree nor disagree) that was combined with “disagree”. A sensitivity analysis was conducted, combining neutral with agree; however, the direction of the findings showed that similar-support remained the same or was higher between 2012 and 2015. Finally, although a majority of the sample showed increased agreement and support for policies to limit sodium across all of the food environments examined between 2012 and 2015, there is very limited knowledge on whether consumers are willing to take action to have sodium reduced in processed foods [3,23].

## 5. Conclusions

The results of this study suggest that, since 2012, there has been substantial agreement and support for actions to limit sodium in commercially-processed and prepared foods, with most consumers ready for actions to lower sodium in foods served in schools, workplaces, and quick-serve restaurants. Moreover, consumer agreement with policies limiting sodium in these environments has increased between 2012 and 2015. In light of recently published sodium-reduction targets and recommendations to reduce sodium across the food industry [24], this analysis provides the public health community with a current view of consumer attitudes and suggests that there may be an increasing trend towards greater support for some of these policies. Future research could examine the role that clinicians and public health agencies play in educating consumers about the need to reduce sodium and how this knowledge may influence changes in consumer attitudes toward broad-based actions and environment-specific policies. It will also be important to understand whether increased support is associated with other changes in consumer behavior to limit sodium in their diet, such as consumer spending on low-sodium products.

## Figures and Tables

**Table 1:** Weighted percentage of respondents on selective demographic characteristics, SS2012 and SS2015.

		Sample *N* (Weighted %)		
Questionnaire and answers	Total	Year 2012	Year 2015	*p*-Value
Total Sample *N*	7845	3926	3919	
Age (years)				
18–30	1187 (22.8)	616 (22.9)	571 (22.6)	
31–50	2773 (34.2)	1471 (35.2)	1302 (33.1)	
≥51	3885 (43.1)	1839 (41.9)	2046 (44.3)	0.18
Gender				
Male	3674 (48.8)	1843 (49.0)	1831 (48.6)	
Female	4171 (51.2)	2083 (51.0)	2088 (51.4)	0.77
Race				
White, non-Hispanic	5886 (67.0)	2939 (67.6)	2947 (66.3)	
Black, non-Hispanic	738 (11.2)	374 (11.3)	364 (11.0)	
Hispanic	808 (14.5)	390 (14.0)	418 (14.9)	
Other, non-Hispanic	413 (7.4)	223 (7.1)	190 (7.7)	0.71
Household income				
<$15,000	698 (9.1)	317 (9.4)	381 (8.8)	
$15,000–$24,999	626 (9.1)	308 (9.2)	318 (9.0)	
$25,000–$39,999	1230 (14.0)	562 (14.4)	668 (13.7)	
$40,000–$59,999	1366 (16.7)	670 (16.8)	696 (16.7)	
≥60,000	3925 (51.1)	2069 (50.3)	1856 (51.8)	0.85
Education level				
HS graduate or less	2691 (41.9)	1241 (42.0)	1450 (41.8)	
Some college	2446 (28.9)	1264 (29.2)	1182 (28.5)	
Bachelor’s degree or higher	2708 (29.2)	1421 (28.8)	1287 (29.7)	0.76
Region				
Northeast	1415 (18.0)	718 (17.7)	697 (18.4)	
Midwest	1984 (21.7)	992 (22.1)	992 (21.4)	
South	2712 (36.8)	1340 (36.8)	1372 (36.8)	
West	1734 (23.4)	876 (23.4)	858 (23.4)	0.86
Body mass index (BMI)				
<25	2774 (37.9)	1433 (38.7)	1341 (37.1)	
25–30	2644 (32.2)	1324 (32.5)	1320 (32.0)	
≥30	2427 (29.9)	1169 (28.8)	1258 (30.9)	0.26
Hypertension Status				
Yes	2162 (25.9)	1097 (27.1)	1065 (24.8)	
No	5683 (74.1)	2829 (72.9)	2854 (75.2)	0.06
Want to eat a diet low in sodium				
Yes	4550 (57.9)	2240 (57.3)	2310 (58.4)	
Neutral	2217 (28.2)	1075 (26.9)	1142 (29.6)	
No	1078 (13.9)	611 (15.8)	467 (12.0)	**0.0004**

HS, High school. SS, SummerStyles. Boldface signifies statistical significance (*p*-Value < 0.05).

**Table 2:** Agreement with broad-based actions to limit sodium in restaurant and manufactured foods, SS2012 and SS2015.

	Agree to Limit Sodium in Restaurant Food			Agree to Limit Sodium in Manufactured Food		
Questionnaire and Answers	Year 2012	Year 2015	*p*-Value	Year 2012	Year 2015	*p*-Value
Total	45.7 (43.6–47.9)	45.9 (44.0–47.7)	0.94	56.5 (54.4–58.6)	56.5 (54.7–58.3)	0.99
Age (years)						
18–30	47.1 (42.1–52.2)	44.3 (39.8–48.8)	0.41	55.9 (50.9–60.9)	55.2 (50.7–59.7)	0.84
31–50	41.2 (37.6–44.7)	43.0 (39.8–46.3)	0.45	52.7 (49.2–56.3)	50.7 (47.4–53.9)	0.40
≥51	48.8 (45.8–51.9)	48.8 (46.3–51.2)	0.97	59.9 (56.9–62.9)	61.5 (59.1–63.8)	0.42
Gender						
Male	43.9 (40.8–47.0)	43.6 (40.9–46.2)	0.86	55.0 (51.9–58.1)	54.1 (51.4–56.8)	0.66
Female	47.5 (44.5–50.4)	48.0 (45.5–50.5)	0.78	57.9 (55.0–60.8)	58.7 (56.3–61.2)	0.65
Race						
White, non-Hispanic	43.0 (40.6–45.4)	41.8 (39.8–43.9)	0.46	54.7 (52.3–57.1)	52.9 (50.9–55.0)	0.27
Black, non-Hispanic	57.3 (50.4–64.2)	58.8 (53.1–64.5)	0.74	67.2 (60.5–74.0)	68.9 (63.6–74.2)	0.71
Hispanic	46.4 (39.8–53.0)	53.7 (48.2–59.1)	0.10	52.6 (46.0–59.2)	61.1 (55.7–66.4)	0.051
Other, non-Hispanic	52.0 (42.6–61.4)	46.5 (38.2–54.9)	0.40	63.8 (54.9–72.8)	60.3 (52.1–68.5)	0.57
Household income						
<$15,000	55.6 (47.9–63.4)	46.7 (40.7–52.6)	0.07	66.3 (59.0–73.6)	56.4 (50.5–62.3)	**0.04**
$15,000–$24,999	45.8 (38.3–53.2)	51.4 (44.9–57.9)	0.27	53.6 (46.2–61.1)	58.0 (51.5–64.4)	0.39
$25,000–$39,999	50.8 (45.1–56.4)	46.9 (42.5–51.4)	0.36	64.0 (58.6–69.4)	60.2 (55.9–64.5)	0.28
$40,000–$59,999	45.4 (40.2–50.5)	49.2 (44.8–53.6)	0.26	55.1 (50.0–60.3)	60.2 (55.9–64.4)	0.14
≥60,000	42.6 (39.7–45.5)	43.4 (40.8–46.0)	0.69	53.4 (50.5–56.4)	54.1 (51.4–56.7)	0.76
Education level						
HS graduate or less	49.5 (45.9–53.2)	46.6 (43.6–49.6)	0.23	58.5 (54.9–62.1)	56.2 (53.3–59.2)	0.34
Some college	44.3 (40.6–47.9)	47.6 (44.3–51.0)	0.18	54.6 (51.0–58.2)	58.3 (55.0–61.6)	0.14
Bachelor’s degree or higher	41.7 (38.1–45.3)	43.0 (39.9–46.2)	0.59	55.4 (51.8–59.1)	55.1 (51.9–58.3)	0.89
Region						
Northeast	52.0 (47.0–56.9)	48.6 (44.3–52.9)	0.32	60.3 (55.5–65.1)	59.6 (55.4–63.9)	0.84
Midwest	41.7 (37.5–46.0)	46.3 (42.6–49.9)	0.11	54.2 (49.9–58.5)	56.1 (52.4–59.7)	0.51
South	45.9 (42.3–49.4)	45.8 (42.7–48.9)	0.97	57.3 (53.8–60.8)	56.9 (53.8–59.9)	0.84
West	44.6 (40.0–49.3)	43.4 (39.5–47.3)	0.69	54.4 (49.8–59.0)	53.8 (49.9–57.8)	0.85
BMI						
<25	44.6 (41.2–48.1)	45.9 (42.8–49.0)	0.60	55.1 (51.6–58.6)	55.7 (52.6–58.8)	0.79
25–30	44.1 (40.4–47.8)	45.2 (42.0–48.3)	0.67	55.5 (51.9–59.2)	55.8 (52.7–58.9)	0.91
≥30	49.1 (45.1–53.0)	46.5 (43.3–49.8)	0.33	59.4 (55.5–63.2)	58.1 (54.9–61.3)	0.61
Hypertension Status						
Yes	53.3 (49.2–57.3)	51.7 (48.3–55.2)	0.57	64.2 (60.3–68.1)	63.1 (59.8–66.4)	0.68
No	42.9 (40.4–45.4)	43.9 (41.8–46.1)	0.56	53.6 (51.1–56.1)	54.3 (52.2–56.5)	0.68
Want to eat a diet low in sodium						
Yes	60.3 (57.6–63.1)	59.9 (57.6–62.3)	0.84	70.9 (68.3–73.4)	71.7 (69.6–73.8)	0.63
Neutral	28.4 (24.7–32.2)	27.0 (23.9–30.0)	0.55	40.9 (36.9–44.9)	35.7 (32.4–38.9)	**0.05**
No	22.4 (17.8–27.0)	23.9 (19.3–28.4)	0.65	30.8 (25.9–35.7)	33.7 (28.7–38.6)	0.43

HS, High school. SS, SummerStyles. Boldface signifies statistical significance (*p*-Value < 0.05).

**Table 3:** Support policies to limit sodium in foods prepared in schools, workplaces, and quick-serve restaurants, SS2012 and SS2015.

	School Cafeterias			Workplace Cafeterias			Quick-Serve Restaurants		
Questionnaire and Answers	Year 2012	Year 2015	*p*-value	Year 2012	Year 2015	*p*-Value	Year 2012	Year 2015	*p*-Value
Total	80.0 (78.3–81.8)	84.9 (83.5–86.2)	<0.0001	71.2 (69.3–73.1)	76.6 (75.1–78.2)	<0.0001	70.8 (68.9–72.7)	76.7 (75.2–78.3)	<0.0001
Age (years)									
18–30	79.0 (74.9–83.1)	81.2 (77.6–84.8)	0.42	68.1 (63.3–72.8)	73.8 (69.8–77.7)	0.07	67.6 (62.8–72.4)	73.5 (69.5–77.5)	0.06
31–50	79.6 (76.7–82.6)	84.0 (81.6–86.4)	**0.03**	70.0 (66.8–73.2)	74.4 (71.5–77.2)	**0.045**	68.7 (65.4–71.9)	73.6 (70.7–76.4)	**0.03**
≥51	81.0 (78.6–83.3)	87.4 (85.8–89.0)	**<0.001**	73.9 (71.3–76.5)	79.8 (77.8–81.7)	**0.0004**	74.3 (71.7–76.9)	80.7 (78.8–82.7)	**<0.0001**
Gender									
Male	77.5 (75.0–80.1)	83.0 (80.9–85.0)	**0.001**	67.4 (64.5–70.2)	73.6 (71.2–75.9)	**0.001**	66.7 (63.8–69.6)	73.4 (71.0–75.8)	**0.0005**
Female	82.5 (80.2–84.7)	86.7 (84.9–88.4)	**0.004**	74.9 (72.4–77.4)	79.5 (77.5–81.6)	**0.005**	74.7 (72.2–77.2)	79.9 (77.9–81.9)	**0.002**
Race									
White, non-Hispanic	78.2 (76.2–80.2)	83.1 (81.5–84.7)	**0.0002**	67.5 (65.3–69.8)	73.9 (72.0–75.7)	**<0.0001**	67.7 (65.5–69.9)	74.3 (72.5–76.1)	**<0.0001**
Black, non-Hispanic	89.6 (85.4–93.8)	90.0 (86.7–93.3)	0.88	83.8 (78.8–88.7)	85.5 (81.4–89.7)	0.59	82.7 (77.4–87.9)	86.2 (82.2–90.1)	0.30
Hispanic	80.6 (75.1–86.1)	88.5 (84.8–92.1)	**0.02**	73.5 (67.5–79.5)	80.3 (75.8–84.7)	0.08	73.0 (67.0–79.0)	80.0 (75.6–84.5)	0.07
Other, non-Hispanic	81.5 (74.4–88.6)	85.7 (79.8–91.5)	0.38	81.4 (74.5–88.3)	80.7 (74.2–87.1)	0.88	77.1 (69.3–84.9)	77.7 (70.7–84.7)	0.91
Household income									
<$15,000	79.8 (73.4–86.1)	80.1 (75.0–85.3)	0.93	72.7 (65.7–79.7)	77.0 (71.9–82.1)	0.33	70.2 (62.9–77.4)	75.0 (69.6–80.3)	0.30
$15,000–$24,999	80.4 (74.2–86.5)	86.3 (81.9–90.8)	0.13	73.9 (67.3–80.6)	77.9 (72.4–83.5)	0.37	74.8 (68.2–81.4)	79.5 (74.3–84.8)	0.28
$25,000–$39,999	83.7 (79.5–87.9)	86.0 (82.9–89.1)	0.40	78.0 (73.4–82.6)	79.3 (75.7–82.9)	0.65	77.7 (73.1–82.4)	80.7 (77.3–84.1)	0.31
$40,000–$59,999	77.3 (72.9–81.6)	87.5 (84.7–90.4)	**0.0001**	73.1 (68.7–77.5)	79.9 (76.5–83.4)	**0.02**	71.1 (66.5–75.7)	79.6 (76.0–83.2)	**0.004**
≥60,000	79.9 (77.6–82.2)	84.3 (82.4–86.2)	**0.004**	67.8 (65.2–70.5)	74.6 (72.3–76.9)	**0.0002**	68.1 (65.4–70.8)	74.6 (72.3–76.8)	**0.0003**
Education level									
HS graduate or less	80.2 (77.2–83.1)	83.0 (80.7–85.3)	0.14	72.9 (69.7–76.2)	75.6 (72.9–78.2)	0.21	73.8 (70.6–77.1)	76.7 (74.1–79.3)	0.17
Some college	79.9 (77.1–82.8)	86.4 (84.2–88.7)	**0.0005**	72.6 (69.5–75.7)	78.7 (76.0–81.4)	**0.004**	72.5 (69.3–75.7)	78.1 (75.3–80.9)	**0.009**
Bachelor’s degree or higher	80.0 (77.0–82.9)	86.0 (83.8–88.2)	**0.001**	67.2 (63.9–70.6)	76.1 (73.5–78.8)	**<0.0001**	64.6 (61.2–68.1)	75.4 (72.7–78.1)	**<0.0001**
Region									
Northeast	82.5 (78.8–86.2)	88.6 (85.8–91.4)	**0.01**	74.3 (70.1–78.5)	82.4 (79.2–85.7)	**0.003**	74.1 (69.9–78.4)	82.5 (79.3–85.7)	**0.002**
Midwest	79.6 (76.3–83.0)	82.8 (80.0–85.6)	0.15	69.4 (65.5–73.3)	75.1 (72.0–78.2)	**0.02**	68.5 (64.5–72.4)	75.4 (72.3–78.5)	**0.007**
South	78.4 (75.3–81.4)	85.0 (82.7–87.2)	**0.0006**	69.8 (66.5–73.1)	76.0 (73.3–78.6)	**0.004**	69.3 (66.0–72.6)	76.2 (73.6–78.9)	**0.001**
West	81.2 (77.7–84.8)	83.7 (80.7–86.7)	0.29	72.8 (68.7–76.8)	74.5 (71.0–78.0)	0.53	72.9 (68.8–76.9)	74.2 (70.6–77.7)	0.63
BMI									
<25	79.4 (76.5–82.2)	86.8 (84.6–89.0)	<0.0001	70.9 (67.8–74.0)	78.2 (75.6–80.9)	**0.0004**	70.3 (67.2–73.5)	79.0 (76.4–81.5)	**<0.0001**
25–30	80.6 (77.8–83.5)	81.9 (79.4–84.4)	0.51	71.1 (67.8–74.4)	73.6 (70.8–76.4)	0.25	70.3 (67.0–73.7)	73.4 (70.6–76.2)	0.17
≥30	80.3 (77.1–83.5)	85.6 (83.4–87.9)	**0.007**	71.7 (68.2–75.2)	77.8 (75.1–80.5)	**0.007**	71.9 (68.4–75.5)	77.5 (74.7–80.2)	**0.02**
Hypertension Status									
Yes	85.4 (82.6–88.3)	87.5 (85.3–89.8)	0.25	78.4 (75.1–81.7)	79.4 (76.6–82.2)	0.64	78.6 (75.4–81.9)	80.1 (77.3–82.8)	0.50
No	78.0 (76.0–80.1)	84.0 (82.4–85.6)	<0.0001	68.5 (66.2–70.8)	75.7 (73.9–77.6)	**<0.0001**	67.9 (65.6–70.2)	75.6 (73.8–77.5)	**<0.0001**
Want to eat a diet low in sodium									
Yes	88.8 (87.0–90.6)	92.6 (91.4–93.9)	**0.0007**	81.4 (79.3–83.6)	85.8 (84.2–87.5)	**0.002**	81.1 (78.9–83.3)	86.0 (84.4–87.7)	**0.005**
Neutral	74.3 (70.8–77.8)	77.8 (74.9–80.7)	0.13	64.8 (61.0–68.6)	68.8 (65.6–72.0)	0.11	64.0 (60.1–67.9)	68.5 (65.3–71.7)	0.08
No	58.1 (52.8–63.4)	64.5 (59.4–69.6)	0.09	45.0 (39.6–50.3)	51.1 (45.8–56.4)	0.11	45.0 (39.6–50.3)	51.6 (46.3–56.9)	0.08

HS, High school. SS, SummerStyles. Boldface signifies statistical significance (*p*-Value < 0.05).

**Table 4:** Adjusted odds ratios (aOR) ^1^ with 95% confidence intervals (CI) on outcome measures, SS2012 and SS2015.

Selected Characteristics	Agree to Limit Sodium in Restaurant Food	Agree to Limit Sodium in Manufactured Food	Support Policies to Limit Sodium in Foods Prepared in School, Workplace, and Quick-Serve Restaurants		
			School Cafeterias	Workplace Cafeterias	Quick-Serve Restaurants
Survey year					
2012	Reference	Reference	Reference	Reference	Reference
2015	0.98 (0.87, 1.11)	0.97 (0.86, 1.10)	**1.37 (1.18, 1.61)**	**1.30 (1.14, 1.49)**	**1.34 (1.17, 1.53)**
Age (years)					
18–30	1.11 (0.93, 1.33)	1.03 (0.86, 1.24)	0.93 (0.74, 1.17)	0.84 (0.69, 1.03)	**0.79 (0.65, 0.97)**
31–50	0.91 (0.79, 1.04)	**0.81 (0.70, 0.93)**	1.01 (0.84, 1.21)	0.89 (0.69, 1.03)	**0.84 (0.71, 0.98)**
≥51	Reference	Reference	Reference	Reference	Reference
Gender					
Male	Reference	Reference	Reference	Reference	Reference
Female	1.08 (0.95, 1.22)	1.06 (0.94, 1.20)	**1.24 (1.06, 1.46)**	**1.34 (1.16, 1.53)**	**1.36 (1.18, 1.56)**
Race					
White, non-Hispanic	Reference	Reference	Reference	Reference	Reference
Black, non-Hispanic	**1.65 (1.33, 2.04)**	**1.59 (1.27, 2.00)**	1.88 (1.36, 2.59)	**2.00 (1.52, 2.62)**	**1.96 (1.48, 2.60)**
Hispanic	**1.32 (1.08, 1.62)**	1.09 (0.90, 1.33)	1.33 (1.01, 1.76)	**1.36 (1.08, 1.72)**	**1.32 (1.05, 1.66)**
Other, non-Hispanic	1.28 (0.97, 1.70)	**1.38 (1.03, 1.84)**	1.07 (0.75, 1.53)	**1.74 (1.24, 2.45)**	**1.41 (1.02, 1.94)**
Household income					
<$15,000	**1.39 (1.10, 1.76)**	**1.47 (1.16, 1.88)**	0.89 (0.66, 1.21)	1.19 (0.91, 1.56)	0.97 (0.74, 1.26)
$15,000–$24,999	1.16 (0.93, 1.46)	1.04 (0.83, 1.30)	1.09 (0.80, 1.49)	1.23 (0.94, 1.61)	1.25 (0.95, 1.64)
$25,000–$39,999	1.19 (0.99, 1.44)	**1.44 (1.19, 1.73)**	1.27 (0.99, 1.62)	**1.51 (1.22, 1.86)**	**1.47 (1.19, 1.81)**
$40,000–$59,999	1.17 (0.99, 1.40)	**1.20 (1.01, 1.43)**	1.04 (0.84, 1.29)	**1.33 (1.09, 1.61)**	1.19 (0.98, 1.44)
≥60,000	Reference	Reference	Reference	Reference	Reference
Education level					
HS graduate or less	**1.25 (1.07, 1.46)**	1.04 (0.89, 1.22)	0.94 (0.77, 1.14)	1.12 (0.95, 1.34)	**1.34 (1.12, 1.59)**
Some college	1.14 (0.98, 1.33)	1.02 (0.88, 1.19)	1.02 (0.84, 1.29)	**1.25 (1.06, 1.47)**	**1.34 (1.12, 1.59)**
Bachelor’s degree or higher	Reference	Reference	Reference	Reference	Reference
BMI					
<25	Reference	Reference	Reference	Reference	Reference
25–30	0.96 (0.83, 1.11)	1.01 (0.87, 1.17)	0.84 (0.69, 1.02)	0.86 (0.73, 1.02)	**0.82 (0.70, 0.98)**
≥30	0.97 (0.83, 1.14)	1.04 (0.89, 1.22)	0.85 (0.69, 1.04)	0.84 (0.71, 1.00)	**0.82 (0.69, 0.98)**
Hypertension Status					
Yes	1.14 (0.99, 1.32)	1.14 (0.98, 1.33)	**1.25 (1.03, 1.53)**	1.16 (0.98, 1.37)	1.18 (0.995, 1.40)
No	Reference	Reference	Reference	Reference	Reference
Want to eat a diet low in sodium					
Yes	**5.04 (4.13, 6.15)**	**5.20 (4.33, 6.24)**	**5.91 (4.79, 7.29)**	**5.37 (4.45, 6.47)**	**5.35 (4.43, 6.45)**
Neutral	**1.27 (1.02, 1.58)**	**1.31 (1.07, 1.59)**	**2.02 (1.65, 2.48)**	**2.20 (1.82, 2.67)**	**2.16 (1.78, 2.62)**
No	Reference	Reference	Reference	Reference	Reference

^1^ Covariates in the models were survey year, age, gender, race, household income, education, BMI, hypertension status, and want to eat a diet low in sodium. HS, High school. SS, SummerStyles. Boldface signifies statistical significance (*p*-Value < 0.05).
